# Chromosome-specific painting provides insights into the karyotype evolutionary direction and trajectory in the genus *Medicago*

**DOI:** 10.1093/hr/uhaf313

**Published:** 2025-11-14

**Authors:** Wei Wang, Yuanbin Zhu, Xia Wu, Zixiang Guo, Qian Zheng, Guangzhen Shi, Yuanhao Li, Wenjun Luo, Fei Wang, Haitao Shen, Sheng Zuo, Quanliang Xie, Hongbin Li, Zhuang Meng

**Affiliations:** Key Laboratory of Oasis Town and Mountain-basin System Ecology of Xinjiang Production and Construction Corps, Key Laboratory of Xinjiang Phytomedicine Resource and Utilization of Ministry of Education, College of Life Sciences, Shihezi University, Shihezi 832003, China; College of Life Sciences, Northwest A&F University, Yangling 712100, China; Key Laboratory of Oasis Town and Mountain-basin System Ecology of Xinjiang Production and Construction Corps, Key Laboratory of Xinjiang Phytomedicine Resource and Utilization of Ministry of Education, College of Life Sciences, Shihezi University, Shihezi 832003, China; Anhui Provincial Key Laboratory of Molecular Enzymology and Mechanism of Major Metabolic Diseases, College of Life Sciences, Anhui Normal University, Wuhu 241000, China; Key Laboratory of Oasis Town and Mountain-basin System Ecology of Xinjiang Production and Construction Corps, Key Laboratory of Xinjiang Phytomedicine Resource and Utilization of Ministry of Education, College of Life Sciences, Shihezi University, Shihezi 832003, China; Key Laboratory of Oasis Town and Mountain-basin System Ecology of Xinjiang Production and Construction Corps, Key Laboratory of Xinjiang Phytomedicine Resource and Utilization of Ministry of Education, College of Life Sciences, Shihezi University, Shihezi 832003, China; Key Laboratory of Oasis Town and Mountain-basin System Ecology of Xinjiang Production and Construction Corps, Key Laboratory of Xinjiang Phytomedicine Resource and Utilization of Ministry of Education, College of Life Sciences, Shihezi University, Shihezi 832003, China; Key Laboratory of Oasis Town and Mountain-basin System Ecology of Xinjiang Production and Construction Corps, Key Laboratory of Xinjiang Phytomedicine Resource and Utilization of Ministry of Education, College of Life Sciences, Shihezi University, Shihezi 832003, China; Key Laboratory of Oasis Town and Mountain-basin System Ecology of Xinjiang Production and Construction Corps, Key Laboratory of Xinjiang Phytomedicine Resource and Utilization of Ministry of Education, College of Life Sciences, Shihezi University, Shihezi 832003, China; Key Laboratory of Oasis Town and Mountain-basin System Ecology of Xinjiang Production and Construction Corps, Key Laboratory of Xinjiang Phytomedicine Resource and Utilization of Ministry of Education, College of Life Sciences, Shihezi University, Shihezi 832003, China; Anhui Provincial Key Laboratory of Molecular Enzymology and Mechanism of Major Metabolic Diseases, College of Life Sciences, Anhui Normal University, Wuhu 241000, China; Key Laboratory of Oasis Town and Mountain-basin System Ecology of Xinjiang Production and Construction Corps, Key Laboratory of Xinjiang Phytomedicine Resource and Utilization of Ministry of Education, College of Life Sciences, Shihezi University, Shihezi 832003, China; Key Laboratory of Oasis Town and Mountain-basin System Ecology of Xinjiang Production and Construction Corps, Key Laboratory of Xinjiang Phytomedicine Resource and Utilization of Ministry of Education, College of Life Sciences, Shihezi University, Shihezi 832003, China; Key Laboratory of Oasis Town and Mountain-basin System Ecology of Xinjiang Production and Construction Corps, Key Laboratory of Xinjiang Phytomedicine Resource and Utilization of Ministry of Education, College of Life Sciences, Shihezi University, Shihezi 832003, China

## Abstract

Divergence in basic chromosome numbers among closely related species is widespread in plants, yet a fundamental question regarding the evolutionary direction of karyotype—whether descending (from higher to lower numbers) or ascending (from lower to higher)—remains contentious. Alfalfa (*Medicago sativa* L.), a key forage crop, displays two basic chromosome numbers (*x* = 8 and *x* = 7) within the genus, and whether this divergence arose through descending evolution from 8 to 7 or the reverse remains unclear. Here, we developed a set of chromosome-specific painting markers capable of tracing chromosomal evolutionary trajectories among *Medicago* species. Comparative cytological analysis of seven accessions (*x* = 8) from the *M. sativa* L. complex revealed conserved chromosomal synteny in both diploid and autotetraploid species, with no detectable interchromosomal rearrangements. In *Medicago polymorpha* (*x* = 7), we discovered that the divergence in basic chromosome numbers (*x* = 7 vs. *x* = 8) resulted from large-scale fission–fusion events involving chromosomes 3, 5, and 6, rather than the simple fusion of chromosomes 3 and 7 as previously published genomic hypotheses. Further supporting evidence from rDNA remodeling and phylogenetic analysis indicates a descending evolutionary pathway, with the ancestral *x* = 8 transitioning to *x* = 7 approximately Mid-Miocene (~12 million years ago). Our results offer new insights into *Medicago* speciation and evolutionary origins, and instantiate a strategy for studying karyotypic evolutionary direction in other plant taxa with similar chromosomal dynamics.

## Introduction

The basic chromosome number (*x*) represents the fundamental genomic composition of a eukaryotic species, and its variation among closely related taxa has been widely documented in plants [1, 2]. For example, in *Arabidopsis thaliana* and related Brassicaceae species, x varies among 5, 6, 7, and 8 [3], while in wild sugarcane (*Saccharum spontaneum*), three cytotypes (*x* = 8, 9, and 10) have been identified [4]. Such variation arises from chromosomal rearrangements (e.g. fissions, fusions) and whole-genome duplication (WGD) followed by diploidization. A key question in karyotype evolution is whether the ancestral basic chromosome number tends to decrease (descending dysploidy) or increase (ascending dysploidy) over time. Since many ancestral plant species are extinct or unknown, reconstructing ancestral karyotypes from extant species is crucial for inferring evolutionary trajectories.

Chromosome painting is one of the most direct methods for reconstructing karyotypic evolutionary relationships in extant species and has been widely applied in both plants and animals [3]. The key to successful chromosome painting lies in obtaining probe markers that can specifically trace individual chromosomes. Traditional chromosome painting probes are typically derived from chromosome sorting, microdissection, and bacterial artificial chromosomes (BACs) [5–7]. However, these methods often required blocking repetitive sequences to achieve hybridization specificity [8]. Additionally, whole-chromosome painting remained technically challenging for plant species with complex genomes due to high repeat content and polyploidy. Recently, the emergence of next-generation oligonucleotide-based probes has resolved the lack of probes for individual chromosome identification in plants with complex genomes [9, 10]. The novel oligo-based probe was designed through genome-directed strategies to specifically trace any chromosomal segment or entire chromosome, and they exhibit broad applicability even in closely related species [11, 12]. This novel technology has been widely applied to research on chromosome evolution in many plant species, such as sugarcane [13], cotton [14], maize [8], wheat [15], and cucumber [16]. Oligo-based chromosome painting has overcome previous limitations, making it a powerful tool for comparative cytogenetics and evolutionary genomics.

Alfalfa (*Medicago sativa* L.), a herbaceous legume (family Leguminosae), is a multifunctional crop widely used for livestock forage, human consumption, and medicinal purposes [17]. Renowned as ‘the queen of forage’ in animal husbandry [18], this species belongs to the genus *Medicago*, which comprises ~87 species with two predominant basic chromosome numbers (*x* = 7 and *x* = 8). Within the genus, chromosome numbers primarily occur as diploids (2*n* = 14, 16), autotetraploids (2*n* = 32), and hexaploids (2*n* = 48), with the tetraploid form being most prevalent [19]. Recent advances have enabled genome sequencing of several *Medicago* species, including *Medicago truncatula* [20], *Medicago ruthenica* [21], *Medicago sativa* ssp. *caerulea* [17, 22], *Medicago sativa* [23], *Medicago falcata* [24], and *Medicago polymorpha* [25]. Despite these genomic resources, the evolutionary trajectory of chromosome number variation within the genus—specifically whether *x* = 7 derived from *x* = 8 through descending dysploidy or vice versa—remains unresolved.

In this study, we developed a comprehensive whole-chromosome painting probe system for all eight chromosomes of *M. sativa*, enabling precise chromosome identification across *Medicago* species. Applying this innovative cytogenetic tool, we analyzed karyotypes of six autotetraploid (*x* = 8) and two diploid (*x* = 7 and *x* = 8) accessions, revealing remarkable chromosomal synteny conservation within the *M. sativa* complex. Our results demonstrate that the divergence between *x* = 7 and *x* = 8 karyotypes resulted from a major chromosomal rearrangement involving chromosomes 3, 5, and 6 through fission–fusion events. By integrating cytogenetic evidence of chromosomal rearrangements, rDNA localization patterns, and phylogenetic analyses, we propose a descending dysploidy model where the ancestral *x* = 8 karyotype gave rise to *x* = 7 through chromosomal fusion. These findings provide crucial insights into the speciation history and genome evolution of the *Medicago* genus, resolving a long-standing question about the directionality of chromosome number evolution in this agriculturally important plant group.

## Results

### Alfalfa chromosome-specific painting based on highly customized oligo probes

**Figure 1:**
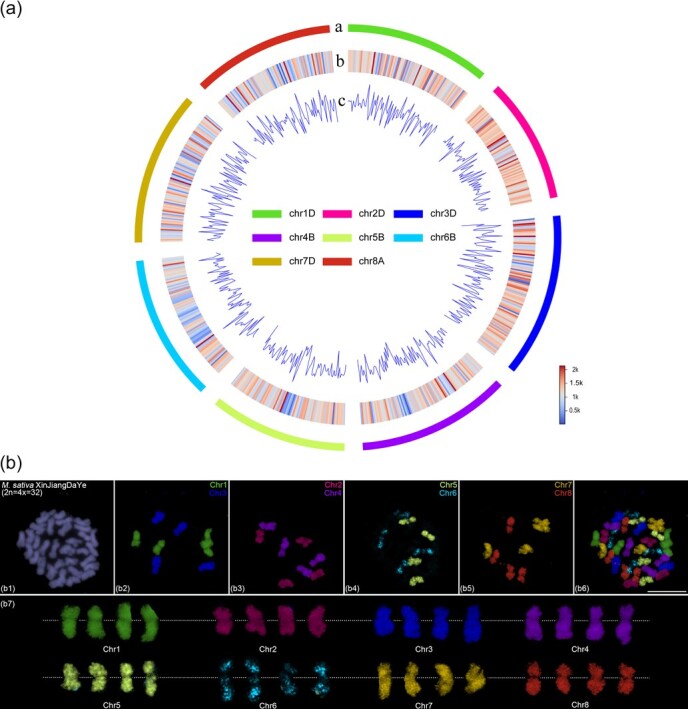
Development of chromosome-specific painting probes based on *M. sativa*. (a) Characteristics of chromosome-specific oligos in the *M. sativa* XinJiangDaYe genome. The outermost track **a** represents the longest homologous copy of each chromosome in *M. sativa* XinJiangDaYe, scaled in megabases (Mb). Heatmap **b** displays the distribution of oligo counts, while the line graph **c** represents oligo density across the entire genome. The number and density of oligos are calculated within 1 Mb windows. (b) FISH assays in *M. sativa* XinJiangDaYe using eight chromosome-specific painting probes. (b1) Metaphase chromosomes counterstained with DAPI. (b2) First-round oligo-FISH using chromosomes 1 and 3-specific painting probes. (b3) Second-round oligo-FISH using chromosomes 2 and 4-specific painting probes. (b4) Third-round oligo-FISH using chromosomes 5 and 6-specific painting probes. (b5) Fourth-round oligo-FISH using chromosomes 7 and 8-specific painting probes. (b6) Each chromosome is represented in different colors using Image-Pro Plus software based on FISH signals from four sequential chromosome painting (b2–b5). (b7) Colored chromosomes were digitally excised from (b6) and magnified 2-fold. White dotted lines indicate centromere positions. Scale bars, 10 μm.

*Medicago sativa* L. (2*n* = 4*x* = 32) is the most widely cultivated and economically valuable forage crop in the world, often referred to as the ‘Queen of Forage’. Previously, we developed barcode probes based on the autotetraploid *M. sativa* ‘Xinjiang Daye’ genome, revealing the sequence basis of presence–absence variation-mediated natural chromosomal aberration tendencies in autotetraploid alfalfa [26]. To further advance research on chromosomal evolution within the *Medicago* genus, we also designed whole-chromosome painting probes for chromosomes 1–8 based on the autotetraploid *M. sativa* ‘Xinjiang Daye’ genome [23]. Using the Chorus2 software [12], we selected the longest pseudomolecule from each of the four homologous copies of chromosomes 1–8 for oligonucleotide probe design, ensuring uniform targeting across all homologous chromosomes. A total of 736 347 chromosome-specific oligos were screened across the eight chromosomes, achieving an average density of ~1 oligo per kilobase (Table S1). Mapping analysis confirmed that the oligos were evenly distributed along each chromosome, with markedly reduced coverage in putative centromeric regions due to highly repetitive sequences (Fig. 1a). Notably, chromosome 6 exhibited a pronounced reduction in oligo density across a large central region, suggesting an accumulation of repetitive elements (Fig. 1a). Further alignment of the oligo sequences to all four homologous copies of each chromosome revealed consistent distribution patterns (Fig. S1), confirming conserved sequence homology and uniform binding efficiency across the autotetraploid subgenomes.

Following polymerase chain reaction (PCR) amplification and fluorescent labeling of the eight chromosome-specific oligo probes, we performed sequential FISH assays to validate their specificity. In each experiment, two probes were hybridized simultaneously, followed by stripping and rehybridization with two additional probes in subsequent cycles. After four rounds of FISH, we successfully visualized all eight chromosome-specific probes within a single cell. The results confirmed that each probe produced strong, specific fluorescent signals across all four homologous chromosomes, demonstrating their suitability for chromosome identification in autotetraploid alfalfa (Fig. 1b). Notably, while probes for chromosomes 1–5, 7, and 8 generated uniform signals along their entire lengths, the chromosome 6 probe exhibited bright fluorescence only at the chromosomal termini, with weak or sparse signals in the interstitial region (Fig. 1b). This observation aligns with our earlier sequence alignment data, further supporting the presence of extensive repetitive sequences in the central region of chromosome 6. Previous chromosome painting-related studies have also reported this phenomenon similar to that observed on chromosome 6 of alfalfa, and demonstrated that the cause of this phenomenon is the presence of a large number of highly repetitive sequences (likely 45S rDNA highly tandemly repeated sequences) in these chromosomal regions [27, 28]. Therefore, we analyzed the sequence composition of chromosome 6. Bioinformatics analysis revealed that regions with high abundance of repetitive sequences on chromosome 6 were not as extensive as observed cytologically. The 45S rDNA also occupied only a very small region (less than 1 Mb) on chromosome 6 (Fig. S5). Subsequent cytological analysis confirmed that the large central region with weak oligo probe signals on chromosome 6 indeed contains a highly tandemly repeated 45S rDNA array (Fig. 4a6–a10). This indicates that the weak oligo signals observed in the large central region of chromosome 6 result from the presence of the highly tandemly repeated 45S rDNA sequence, which was not well assembled in the genome, rather than being caused by low probe efficiency.

### Conserved chromosomal collinearity in the *M. sativa* L. complex

**Figure 2:**
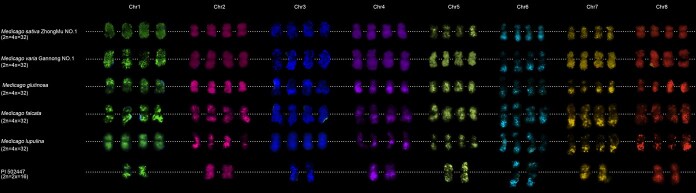
FISH analysis in five autotetraploid *M. sativa* L. complex accessions and one diploid accession using chromosome-specific painting probes. Colored chromosomes were digitally excised from the same images of *M. sativa* ZhongMu No. 1 (Fig. S2a), *M. varia* GanNong No. 1 (Fig. S2b), *M. glutinosa* (Fig. S2c), *M. falcata* (Fig. S2d), *M. lupulina* (Fig. S2e), and PI 502447 (Fig. S3) with 2× magnification. White dotted lines indicate centromere positions.

The *M. sativa* L. complex represents the most important forage resource in the genus *Medicago*. Despite their high morphological, ecological, and genetic similarity, these species exhibit remarkable diversity in chromosome number and ploidy level, ranging from diploid to polyploid. Natural hybridization among them facilitates the formation of complex gene exchange networks. To investigate whether these accessions share conserved chromosomal features, we performed comparative FISH analysis using our chromosome-specific painting probes on five autotetraploid accessions (*M. sativa* Zhongmu No. 1, *Medicago varia* Gannong No. 1, *Medicago glutinosa*, *M. falcata*, and *Medicago lupulina*) and one diploid accession. All eight probes produced strong, specific fluorescence signals in both the autotetraploid (Fig. S2) and diploid (Fig. S3) accessions. No interchromosomal translocations or rearrangements were detected, indicating a high degree of synteny conservation across the *M. sativa* L. complex (Fig. 2). We hypothesize that this conserved chromosomal organization facilitates the production of fertile hybrids through natural hybridization.

### Dramatic chromosomal breakage and fusion in *M. polymorpha*

**Figure 3:**
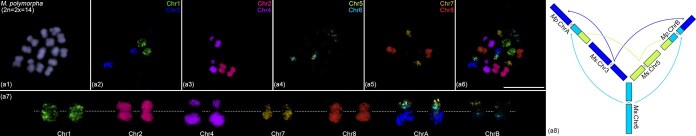
Chromosome painting in *M. polymorpha* (2*n* = 2*x* = 14)*.* (a1) Metaphase chromosomes counterstained with DAPI. (a2–a5) Four-round FISH assays in *M. polymorpha* using chromosome-specific painting probes. (a6) Each chromosome is represented in different colors using Image-Pro Plus software based on FISH signals from four sequential chromosome painting (a2–a5). (a7) Colored chromosomes were digitally excised from (a6) and magnified 2-fold. White dotted lines indicate centromere positions. (a8) An ideogram illustrating chromosomal rearrangement events involving chromosome 3, 5, and 6 between *M. sativa* (*Ms*.) and *M. polymorpha* (*Mp*.) based on FISH results. Scale bars, 10 μm.

Previous genomic studies identified *M. polymorpha* (2*n* = 14) as a diploid species whose chromosome 3 originated from the fusion of chromosomes 3 and 7 of *M. sativa* [25]. To cytologically validate these findings, we performed FISH analysis in *M. polymorpha* using chromosome-specific painting probes designed from *M*. *sativa*. FISH analysis revealed differential chromosomal evolution between the species: while probes for chromosomes 1, 2, 4, 7, and 8 showed highly conserved hybridization patterns (suggesting synteny retention), those for chromosomes 3, 5, and 6 exhibited substantial signal redistribution, pointing to complex rearrangements (Fig. 3a1–a7). Cytological analysis revealed that chromosomes 3, 5, and 6 underwent fragmentation into two segments, respectively. These segments subsequently reassembled into two novel chromosomes (ChrA and ChrB) following a consistent structural pattern: the broken segments of chromosomes 3 and 5 formed the distal regions, while the fragmented segments of chromosome 6 occupied the central region (Fig. 3a8). Our findings reveal that the reduced chromosome number in *M. polymorpha* resulted not from a simple fusion of chromosomes 3 and 7, but rather from extensive fission–fusion events involving three ancestral chromosomes (3, 5, and 6), ultimately generating two new composite chromosomes.

### rDNA remodeling and chromosomal rearrangement confirmed descending dysploidy in alfalfa

**Figure 4:**
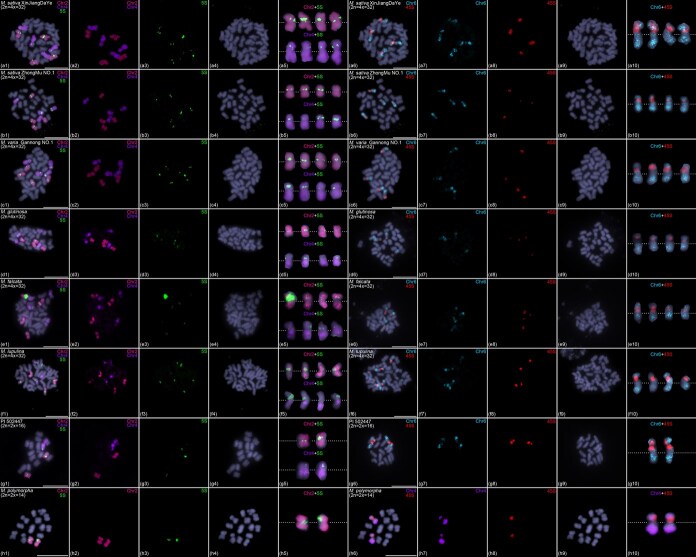
Chromosomal distribution of 5S and 45S rDNA in eight *Medicago* accessions. FISH analysis of 5S and 45S rDNA in *M. sativa* XinJiangDaYe (a1–a10), *M. sativa* ZhongMu No. 1 (b1–b10), *M. varia* GanNong No. 1 (c1–c10), *M. glutinosa* (d1–d10), *M. falcata* (e1–e10), *M. lupulina* (f1–f10), PI 502447 (g1–g10), and *M. polymorpha* (h1–h10). (a–g) FISH results showed that 5S rDNA was located on chromosomes 2 and 4, while 45S rDNA was located on chromosome 6 in seven *Medicago* accessions with *x* = 8. (h) In *M. polymorpha* (*x* = 7), 5S rDNA was only located on chromosomes 2, while 45S rDNA was located on chromosome 4. Colored chromosomes were digitally excised from (a1–h1, a6–h6) and magnified 2-fold. White dotted lines indicate centromere positions. Scale bars, 10 μm.

Ribosomal DNA (rDNA), comprising the highly conserved 45S and 5S multigene families that encode essential ribosomal RNA components, serves as a critical molecular marker for investigating species origins and evolutionary relationships. To elucidate the evolutionary trajectory of rDNA organization in *Medicago* species, we conducted FISH analysis using 45S and 5S rDNA probes across eight representative alfalfa accessions. FISH results showed that 5S rDNA resided on the four homologous copies of chromosomes 2 and 4, respectively, whereas 45S rDNA was precisely located in low oligo signal interstitial region on the four homologous copies of chromosomes 6 in six autotetraploid alfalfa (2*n* = 4*x* = 32) accessions (i.e. *M. sativa* Xinjiang Daye, *M. sativa* Zhongmu No. 1, *M. varia* Gannong No. 1, *M. glutinosa*, *M. falcata*, and *M. lupulina*) (Fig. 4a–f). The results suggest that the unknown repetitive sequences within the low-oligo-signal interstitial regions on chromosome 6 may correspond to 45S rDNA clusters. Interestingly, our FISH results showed that the signal of Chr6 and 45S were both weak in *M. glutinosa* and *M. falcata*. Previous oligo-FISH-related studies have demonstrated that probe signal intensity positively correlates with phylogenetic relatedness among species [29, 30]. Consequently, the weaker signals of chromosome 6-specific oligo probes in *M. glutinosa* and *M. falcata* may suggest a greater evolutionary distance between *M. glutinosa*/*M. falcata* and *M. sativa* species. We speculate that the chromosome 6-specific oligo sequences selected from *M. sativa* may exhibit significant sequence variation compared to the corresponding regions in *M. glutinosa* and *M. falcata*, resulting in incomplete hybridization of these oligos to chromosome 6 in these two species and thereby producing weaker signals. For the weak 45S rDNA signals, it is well known that highly tandemly repeated rDNA sequences evolve rapidly, even exhibiting significant copy number variation, positional shifts, or loss among different species within the same genus [31]. Therefore, the weaker 45S rDNA signals observed in *M. glutinosa* and *M. falcata* compared to other *Medicago* species may be attributed to a reduction in 45S rDNA copy numbers during their evolutionary history. In diploid alfalfa (2*n* = 2*x* = 16), we observed a conserved chromosomal distribution pattern of rDNA similar to that in autotetraploid alfalfa, with 5S rDNA located on chromosomes 2 and 4, and 45S rDNA on chromosome 6 (Fig. 4g1–g10). These results demonstrate remarkable evolutionary conservation of rDNA chromosomal organization across different ploidy levels in *Medicago* species with a base chromosome number of *x* = 8, highlighting the stability of these genomic regions during polyploidization events and species diversification.

Notably, our cytological investigations in diploid *M. polymorpha* (2*n* = 2*x* = 14) revealed significant rDNA reorganization events: (i) complete elimination of the ancestral 5S rDNA locus on chromosome 4 with exclusive retention on chromosome 2; (ii) translocation of 45S rDNA from its ancestral position on chromosome 6 to chromosome 4 following chromosomal fission–fusion events (Fig. 4h1–h10). These dramatic structural modifications prompt a fundamental evolutionary question regarding the directionality of chromosome number change in *Medicago*—whether through reduction from ancestral *x* = 8 to derived *x* = 7, or alternatively via increase from ancestral *x* = 7 to derived *x* = 8.

**Figure 5:**
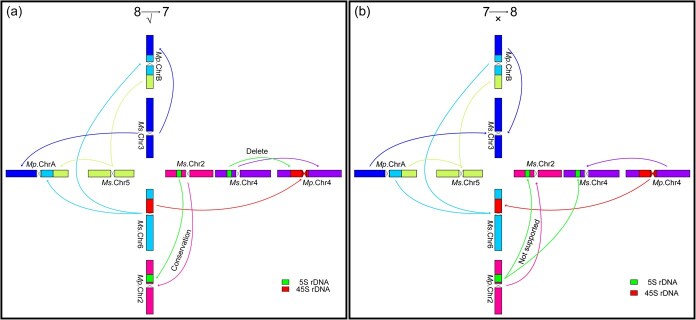
Schematic diagram of two hypothesized evolutionary histories for basic chromosome number divergence in the genus *Medicago*. (a) An ideogram illustrating the evolutionary model for basic chromosome number reduction (*x* = 8 → 7) in the genus *Medicago.* This hypothesis is supported by cytogenetic evidence from rDNA reorganization and chromosomal rearrangements. (b) An ideogram illustrating the evolutionary model for the increase in basic chromosome number reduction (*x* = 7 → 8) in the genus *Medicago* (lacking cytological evidence support). The schematic diagram was drawn based on the cytological results of rDNA restructuring and chromosomal rearrangements. *Medicago sativa* (*x* = 8, *Ms*.) and *M. polymorpha* (*x* = 7, *Mp*.).

Our comprehensive cytogenetic evidence strongly supports a descending evolutionary model (*x* = 8 → *x* = 7) based on three key observations: First, the reduction model presents a more parsimonious and mechanistically plausible scenario. This pathway would require: (i) fragmentation of chromosomes 3, 5, and 6 into two segments each; (ii) subsequent fusion of these fragments into two novel chromosomes; and (iii) concurrent loss of 5S rDNA from chromosome 4 with acquisition of 45S rDNA derived from chromosome 6 fission–fusion events (Fig. 5a). This straightforward process aligns perfectly with our cytological observations. Second, the alternative ascending model (*x* = 7 → *x* = 8) demands substantially more complex chromosomal rearrangements that contradict our empirical findings. This hypothetical scenario would necessitate: (i) fragmentation of two chromosomes into three segments; (ii) reorganization of these fragments into three new chromosomes; (iii) transfer of 45S rDNA from chromosome 4 to the newly formed chromosome 6; and (iv) simultaneous acquisition of partial 5S rDNA by chromosome 4 from chromosome 2 (Fig. 5b). Crucially, this model predicts structural modifications to chromosome 2 that are entirely absent in our cytological data. Third, the exceptional evolutionary conservation of chromosome 2 across both *x* = 8 and *x* = 7 *Medicago* species provides compelling negative evidence against the ascending model. The complete absence of detectable structural aberrations in chromosome 2 throughout the evolutionary process further reinforces the descending model as the most plausible scenario for chromosome number evolution in *Medicago*.

### Comparative karyotyping of *Medicago* species based on individually identified chromosomes

**Figure 6:**
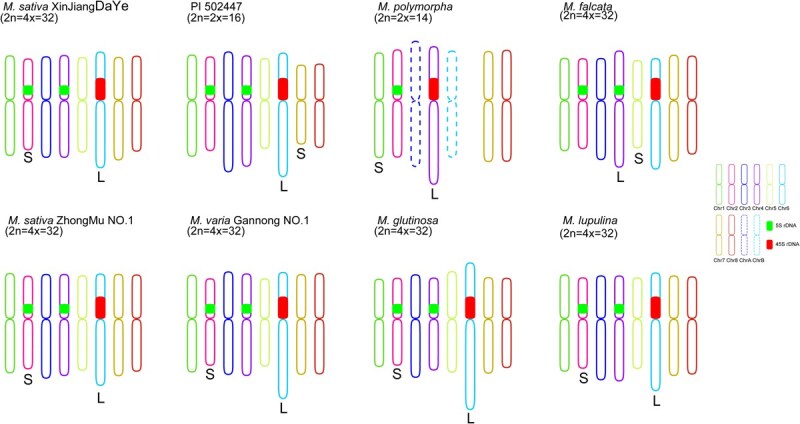
Comparative karyotype analysis among eight *Medicago* accessions. Schematic illustration of chromosome karyotype in eight Medicago accessions. L and S denote the longest and shortest chromosomes of each *Medicago* species, respectively. The relative length and arm ratio of each chromosome, as well as the location of rDNA, were drawn based on the FISH results and karyotype data (Table S3).

Our research demonstrated that the developed whole chromosome painting probe can distinguish and identify each chromosome in *Medicago* species. Based on cytological results, we established chromosomal karyotypes for these eight *Medicago* accessions, including autotetraploid *M. sativa* Xinjiang Daye, *M. sativa* Zhongmu No. 1, *M*. *varia* Gannong No. 1, *M. glutinosa*, *M. falcata*, *M. lupulina*, diploid PI 502447, and *M. polymorpha* (Table S3). The karyotype results revealed that nearly all chromosomes were metacentric among the eight alfalfa accessions (Table S3). Comparative karyotypic analysis revealed that the karyotypes of five autotetraploid alfalfa accessions (i.e. *M. sativa* Xinjiang Daye, *M. sativa* Zhongmu No. 1, *M*. *varia* Gannong No. 1, *M. glutinosa* and *M. lupulina*) were highly similar: their longest chromosome was chromosome 6, and the shortest was chromosome 2 (Table S3, Fig. 6). The published genome of *M. sativa* Xinjiang Daye [23] reported chromosome 2 (76.8 Mb) as the shortest, which aligns with our cytological findings. However, the genome assembly identified chromosome 3 (100.4 Mb)—rather than chromosome 6 (89.6 Mb)—as the longest, contradicting our cytogenetic results. Indeed, discrepancies between cytologically measured chromosome lengths and pseudomolecule sizes in genome assemblies have been documented in other plant species, such as chromosome 2 in tomato [29] and chromosome 8 in licorice [28]. In both cases, the presence of highly tandemly repeated 45S rDNA arrays resulted in underestimated pseudomolecule sizes relative to their true cytological lengths. Although third-generation sequencing technologies have significantly improved read lengths, accurate assembly of large-spanning repetitive sequences like 45S rDNA remains challenging [32]. We have already analyzed the assembly status of 45S rDNA in the current genome, and found that only a minor fraction of 45S rDNA (less than 1 Mb) was successfully assembled to chromosome 6 (Fig. S5). However, cytological analysis revealed that 45S rDNA occupies a large central region of chromosome 6 (occupies ~1/4 of the entire chromosome) (Fig. 4a6–a10). Based on our genomic and cytological analyses data, we propose that misassembly of 45S rDNA may be the primary cause of the inconsistency between the genomic length and true cytological length of chromosome 6. In *M. falcata*, we observed substantial karyotype divergence compared to the other five autotetraploid *Medicago* accessions, with the longest and shortest chromosomes having shifted to chromosomes 4 and 5, respectively (Table S3, Fig. 6).

In diploid accession (2*n* = 2*x* = 16), we observed that their karyotypes are not highly similar to those of autotetraploid alfalfa (with the longest and shortest chromosomes being chromosomes 6 and 7, respectively) (Table S3, Fig. 6), suggesting that karyotypes may have undergone variations during WGD. In *M. polymorpha* (2*n* = 2*x* = 14), we observed dramatic karyotypic restructuring following chromosomal rearrangement. Chromosome 4 became the longest chromosome due to the integration of the 45S rDNA locus, while chromosome 1 became the shortest (Table S3, Fig. 6). Subsequently, we conducted a comparative significant difference analysis to further investigate the evolutionary characteristics of rDNA among the eight alfalfa accessions, as well as the arm ratios and relative lengths of chromosomes 1–8. The results revealed significant differences in arm ratios (excluding chromosome 1), relative lengths, and rDNA across all chromosomes among the eight materials (Fig. S4), indicating karyotypic evolutionary diversity in the genus *Medicago*.

### Reconstruction of alfalfa’s evolutionary framework origin from ancestors based on phylogenetic and cytogenetic analysis

**Figure 7:**
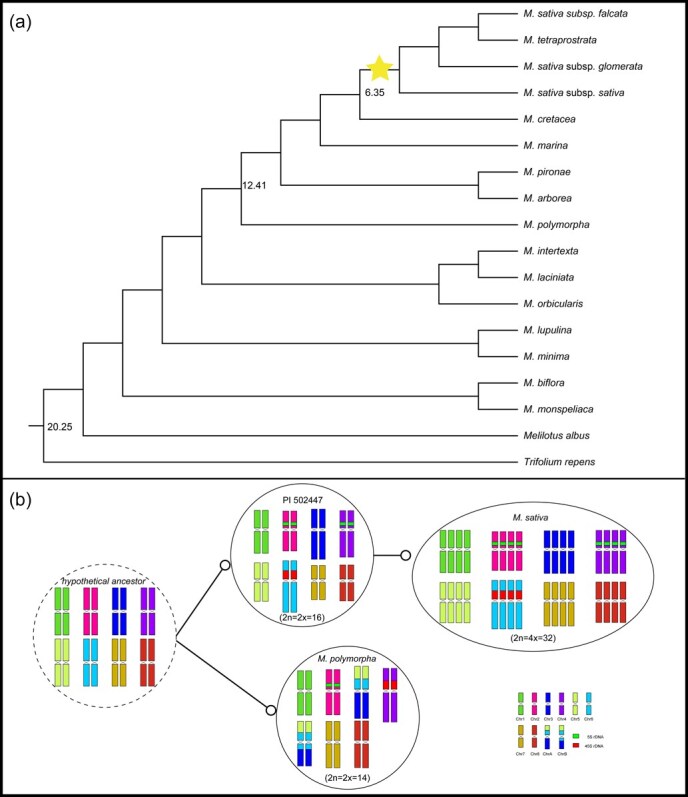
Inferred evolutionary framework of the *Medicago* species. (a) An ML phylogeny based on concatenated 74 plastid PCGs. The star indicates the species-specific auto-tetraploidization. Numbers within the tree represent divergence-time estimates based on MCMC tree analysis. (b) An ideogram illustrating a hypothetical ancestor (*x* = 8) underwent chromosome fission–fusion events, resulting in *M*. *polymorpha* (*x* = 7). Extant diploid *Medicago* species (*x* = 8) retained a similar karyotype to the hypothetical ancestor, while extant autotetraploid alfalfa (*x* = 8) originated via WGD of a diploid progenitor. The schematic illustration was drawn based on comparative chromosome painting results.

To elucidate the timing of chromosome number evolution in *Medicago*, we used MCMCTree software to estimate divergent times. Based on the plastome phylogeny and two secondary calibration points (Fig. 7a), the split between *M. polymorpha* and *M. sativa* was inferred to have diverged during the Mid-Miocene (~12 Mya) (highest posterior density interval = 9.78–14.59) during the Late Miocene (Tortonian). The diploid ancestor of alfalfa underwent autotetraploidization ~6 Mya (Fig. 7a). Moreover, we found that the *Medicago* genus underwent episodes of rapid diversification between ~2 and 4 Mya, leading to the successive divergence of the tetrapolyploids (Fig. 7a).

Based on the findings from cytological and phylogenetic analyses, we reconstructed the evolutionary framework for the origin of *Medicago* species from ancestral to the extant species (Fig. 7b). We propose that *M. polymorpha* (*x* = 7) diverged from a putative ancestral diploid species (*x* = 8) ~12 Mya and subsequently underwent independent evolution. The extant diploid (*x* = 8) may retain a similar chromosomal karyotype of the putative progenitor. Subsequently, this diploid lineage experienced a WGD event, leading to the formation of an autotetraploid species ~6 Mya. During the period of ~2–4 Mya, this autotetraploid *Medicago* lineage underwent rapid diversification, forming diverse lineages and becoming the dominant population.

## Discussion

As the world’s most important legume forage crop, alfalfa (*M. sativa* L.) holds exceptional ecological and economic value, earning its reputation as the ‘Queen of Forage’ [18, 33]. The genus *Medicago* displays remarkable chromosomal diversity that has long fascinated geneticists and evolutionary biologists. This diversity is exemplified by the coexistence of diploids with a basic chromosome number of *x* = 7 (2*n* = 14) alongside diploids (2*n* = 16), tetraploids (2*n* = 32), and hexaploids (2*n* = 48) with *x* = 8 [19, 34, 35]. Such complex karyotypic variation has posed significant challenges for molecular cytogenetic research in *Medicago*, creating a notable gap in our understanding compared to other economically important crops. Previous attempts to overcome these challenges have employed various cytogenetic tools, including genomic DNA probes [36], repetitive sequence markers [37], BAC clones [38], and more recently, oligo-based barcode probes [26]. While these approaches have undoubtedly advanced alfalfa cytogenetics, they have inherent limitations that restrict their utility. Genomic DNA probes, while useful for general karyotyping, fail to provide chromosome-specific information. Similarly, repetitive sequence markers and BAC clones can only identify partial chromosomal regions rather than enable comprehensive whole-chromosome analysis. These technical constraints have significantly hampered progress in understanding the chromosomal basis of *Medicago* evolution and diversification. Our whole-chromosome painting probe system enables accurate tracing of chromosomal characteristics and evolutionary trajectories in *Medicago* species (Figs 1–3), providing a reliable tool for future studies on genome evolution and speciation within *Medicago* plant groups. Furthermore, this system holds significant application potential in germplasm monitoring, karyotype stability testing, and hybrid identification. In germplasm monitoring, it can rapidly identify chromosomal composition differences among various *Medicago* germplasm resources, aiding in precise classification and genetic integrity evaluation of germplasm repositories. For karyotype stability testing, it enables visualization of chromosomal structures to monitor karyotypic characteristics after multiple generations of propagation, thereby assessing genotypic stability. In hybrid identification, it can track the transmission of parental chromosomes from *Medicago* species in distant hybrid offspring, confirming hybridization success and chromosomal origins to enhance breeding efficiency. We anticipate that this technology will serve as a cornerstone for future studies in *Medicago* cytogenetics and related fields.

### Chromosomal conservation and evolution in the *M. sativa* complex

The *M. sativa* complex, which includes cultivated alfalfa and its closely related wild relatives, represents the most agriculturally valuable group within the genus *Medicago* [19, 39]. This complex is particularly intriguing to evolutionary biologists due to its ability to maintain genetic cohesion despite comprising both diploid and autotetraploid forms that freely hybridize in nature. Our comprehensive analysis of six autotetraploid accessions (including three *M. sativa* varieties, *M. glutinosa*, *M. falcata*, and *M. lupulina*) and one diploid accession has revealed an extraordinary degree of chromosomal collinearity conserved across evolutionary timescales (Fig. 2 and Figs S2, S3). This conservation is evident at multiple levels. First, our cytogenetic data show no detectable interchromosomal rearrangements among these taxa. Second, these findings are supported by previous studies demonstrating high similarity in repetitive sequence organization between diploids and tetraploids [39]. Third, recent genome sequencing projects have confirmed that wild diploid progenitors exhibit remarkable synteny with cultivated tetraploid varieties [17, 22]. The maintenance of such chromosomal stability despite polyploidization and speciation events suggests strong selective pressures preserving genome architecture in this group. Recent *Medicago* pan-genome studies revealed strong collinearity among the genomes of ZM4 (tetraploid), *M. sativa* spp. *Caerulea* (diploid), and *M. truncatula* genotype Jemalong A17 (diploid). Additionally, chromosomal-level assemblies for 24 alfalfa accessions not only exhibited similar chromosome sizes, but also demonstrated a high degree of synteny [40]. These results are consistent with our findings of conserved chromosomal collinearity during the evolution of the *M. sativa* Complex, further supporting our related hypotheses. We hypothesize that this remarkable karyotypic conservation may serve several important biological functions. First, it likely facilitates ongoing natural hybridization between cytotypes, maintaining genetic connectivity across ploidy levels. Second, it may contribute to genome stability by minimizing meiotic errors in polyploids. Third, it could preserve favorable gene linkages that have been maintained by selection. The exception to this pattern is *M. falcata*, which shows distinct karyotypic features including significant variation in chromosome sizes (Fig. 6, Table S3). This divergence suggests that particular evolutionary forces may be operating differently in this lineage, presenting an intriguing avenue for future research.

### Mechanisms of chromosomal rearrangement and speciation in the genus *Medicago*

Chromosomal rearrangements have long been recognized as fundamental drivers of speciation and evolutionary diversification [41, 42]. In *Medicago*, the diploid species *M. polymorpha* (2*n* = 14) has attracted particular attention due to its derived chromosome number compared to most congeners (2*n* = 16 or 32) [43, 44]. Previous genomic analysis suggested a relatively simple fusion event between chromosomes 3 and 7 as the mechanism for chromosome number reduction [25]. However, our detailed cytogenetic investigation reveals a more complex evolutionary scenario involving coordinated rearrangements of chromosomes 3, 5, and 6 (Fig. 3). We speculate that the inconsistency between our cytological results and the previously published genomic hypotheses may be explained by several plausible explanations. (i) Genome assembly challenges: Complex, highly repetitive centromeric and pericentromeric regions—which are often misassembled in short-read genome projects—may be a potential cause of artificially inferred chromosomal fusions and incorrect scaffolding. (ii) Methodological differences: We highlight the fundamental differences between *in silico* genomic inference and direct cytogenetic observation. Cytogenetics provides a physical map of the chromosomes as they exist *in vivo*, while genomic assembly is an interpretive reconstruction that can be confounded by biological complexities like repeats and heterozygosity. (iii) Biological variation: We also consider the possibility that the accession studied by Cui *et al.* and ours, though both *M. polymorpha*, might represent different lines or ecotypes with genuine karyotypic variation. However, we additionally examined the chromosomal composition of *M. polymorpha* L. ‘Chuxiong’ (2*n* = 2*x* = 14) and found it consistent with the cytological data of *M. polymorpha* presented in our manuscript. We note that this is considered less likely than technical explanations unless future studies confirm it. Genomic analyses are powerful for generating hypotheses, while cytogenetic validation remains a critical step for confirming chromosomal rearrangements. Our direct observational data therefore present a strong challenge to the ‘3 + 7 fusion’ hypothesis and suggest that the genomic assembly might require re-evaluation, particularly in the pericentromeric regions of the chromosomes in question. Our findings significantly advance our understanding of karyotype evolution in several important ways. First, it demonstrates that chromosome number changes can involve multiple concurrent rearrangements rather than single fusion events. Second, it highlights the importance of combining modern genomic data with classical cytogenetic approaches to fully understand chromosomal evolution. Third, it provides a concrete example of how chromosomal rearrangements can contribute to reproductive isolation and speciation in plants. Furthermore, we reconstructed the speciation phylogeny of *M. polymorpha* and other *Medicago* species based on plastome data with secondary calibration points, and clarified that the divergence between *M. polymorpha* (*x* = 7) and other *Medicago* species (*x* = 8) occurred approximately in the Mid-Miocene (~12 million years ago), while diploid *Medicago* autopolyploidization events were dated to ~6 million years ago. By integrating phylogenetic tree and cytogenetic data, we reconstructed the karyotype evolutionary trajectory of these *Medicago* species, providing a valuable reference framework for studies on speciation and chromosomal evolution in the genus. However, it should be noted that divergence time estimations based solely on plastome data can be influenced by its specific mode of inheritance and the potential for historical introgression, which may not always reflect the true species divergence history. Although the plastome phylogeny provides a robust hypothesis of maternal lineage relationships, it may not represent the complete species history in scenarios involving reticulate evolution. We propose that future research employing high-throughput sequencing of multiple nuclear loci (e.g. target enrichment or whole genome sequencing) would be invaluable. Such nuclear genomic data could be used to: (i) reconstruct a species tree and assess potential discordance with the plastome topology presented here; (ii) explicitly test for historical introgression events and estimate their direction and intensity using phylogenetic network analyses; and (iii) refine our understanding of divergence times and evolutionary relationships within *Medicago* by leveraging the independent evidence from the biparentally inherited nuclear genome.

### Resolving the evolutionary direction of basic chromosome number in alfalfa

Divergence in the basic chromosome number among closely related species has been extensively documented in diverse plant taxa [45–48]. The coexistence of *x* = 7 and *x* = 8 cytotypes in *Medicago* presents a classic evolutionary puzzle that has remained unresolved despite decades of research [49]. Our study provides multiple lines of evidence supporting a descending dysploidy model (*x* = 8 → *x* = 7) as the most plausible evolutionary trajectory. This conclusion is based on three key findings: First, our analysis of chromosomal rearrangement patterns reveals that the transition can be explained by a single fragmentation event followed by reciprocal fusions (Fig. 3). This mechanism is evolutionarily parsimonious compared to alternative scenarios requiring multiple independent rearrangements. Second, we observed coordinated changes in rDNA localization patterns accompanying the chromosomal rearrangements (Fig. 4). In *x* = 8 species, 45S rDNA is located on chromosome 6 while 5S rDNA sites are found on chromosomes 2 and 4. In contrast, *x* = 7 *M. polymorpha* shows relocation of 45S rDNA to chromosome 4 and reduction of 5S rDNA to a single site on chromosome 2. These changes are most easily explained by the descending model. A key question is whether the movement of 45S rDNA from chromosome 6 to chromosome 4 is accompanied by any other sequences from chromosome 6. Theoretically, we speculate that the translocation of 45S rDNA from Chr6 to Chr4 without any accompanied sequences seems unlikely. Such a transfer should be accompanied by some sequences from chromosome 6. However, oligo probes require a certain quantity and density to generate detectable signals. If the accompanying chromosome 6 sequences are limited in amount or confined to small regions, the oligo probes may not produce cytologically visible FISH signals. Additionally, the resolution of metaphase chromosomes is relatively low (approximately at the Mb level), making cytological visualization of this process extremely challenging. We believe that further exploration of this question may require assembling a telomere-to-telomere (T2T) genome of *M. polymorpha*, and sequence alignment analyses based on such a high-quality assembly should help resolve this issue. Third, chromosome 2 shows complete conservation between *x* = 7 and *x* = 8 cytotypes, with no structural variation and only quantitative reduction in 5S rDNA. This pattern strongly supports chromosome number reduction rather than increase. To further confirm the descending dysploidy model, we performed FISH assays on another 2*n* = 2*x* = 16 (*M. truncatula* R108) and 2*n* = 2*x* = 14 (*M. polymorpha* L. ‘Chuxiong’) plants using 8 chromosome painting probes. The results were consistent with those previously observed in the diploid PI 502447 and *M. polymorpha*, respectively (Fig. S6). However, given the still-limited sample size of diploid *Medicago* materials (2*n* = 16 and 2*n* = 14), we cannot rule out the existence of other karyotypes in diploid *Medicago* species. Therefore, the descending dysploidy model applies specifically to these *Medicago* species and may not represent the general evolutionary model for the transition from *x* = 8 to *x* = 7 across the entire genus *Medicago*. The evolutionary trajectory we propose finds parallels in other plant systems. In Brassicaceae, for instance, *Arabidopsis thaliana* and its relatives demonstrate how chromosome fission–fusion events and reciprocal translocations can drive reductions in basic chromosome number from *x* = 8 to *x* = 5 [3]. Similarly, in *S. spontaneum*, sequential reductions from *x* = 10 to *x* = 9 and then to *x* = 8 have been documented through similar mechanisms [4, 50]. These consistent patterns across distantly related taxa suggest that descending dysploidy may be a common evolutionary pathway in plants.

## Conclusion

Our study makes several significant contributions to understanding *Medicago* genome evolution. First, we have developed and validated a comprehensive whole-chromosome painting system that overcomes previous technical limitations in *Medicago* cytogenetics. Second, we have demonstrated remarkable karyotypic stability within the *M. sativa* complex despite its complex ploidy variation. Third, we have resolved the long-standing question regarding the evolutionary direction of basic chromosome number in the genus, providing strong evidence for a descending dysploidy model (*x* = 8 → *x* = 7) mediated by complex chromosomal rearrangements. In summary, our study provides both the tools and the conceptual framework to further explore these fascinating evolutionary processes, contributing to an enhanced understanding of the speciation history and genome evolution within the genus *Medicago*.

## Materials and methods

### Plant materials and chromosome preparation

Eight alfalfa accessions were used in this study, including *M. sativa* XinJiangDaYe, *M. sativa* ZhongMu No. 1, *M. varia* GanNong No. 1, *M. glutinosa* (previously published study records that *M. glutinosa* is an autotetraploid (*x* = 8) [51]), *M. falcata*, *M. lupulina*, *M. sativa* subsp. *falcata* (PI 502447), and *M. polymorpha*. Among them, existing studies and our cytological results demonstrate that the first six *Medicago* species in this study are autotetraploid (*x* = 8), while PI 502447 (*x* = 8) and *M. polymorpha* (*x* = 7) are both diploids. These eight alfalfa accessions were all obtained from Professor Quanwen Dou, the Northwest Institute of Plateau Biology, Chinese Academy of Sciences, and were cultivated in the greenhouse of Shihezi University, Xinjiang, China. Metaphase chromosomes were prepared from root tips using the previously described method [26]. Briefly, root tips were collected from eight alfalfa accessions and treated with cycloheximide for 2 h to accumulate metaphase cells, and then fixed in Carnoy’s fixative and stored at −20°C. An enzymatic solution with 4% cellulase (w/v) (Yakult Pharmaceutical, Tokyo, Japan), 2% pectinase (w/v) (Plant Media, Pittsburgh, PA, USA), and 2% (w/v) cellulase ‘Onozuka’ RS (Yakult Pharmaceutical, Tokyo, Japan) was used to digest the root tips for 2 h at 37°C. Subsequently, metaphase chromosomes were prepared following the published protocol [26]. Chromosome slides were stored at −80°C for subsequent FISH assays.

### Development of whole chromosome-specific probes in alfalfa

The reference genome of autotetraploid alfalfa ‘XinJiangDaYe’ [23] was used to design oligo-based chromosome-specific painting probes based on the previously described method [26]. Briefly, the longest homologous copy among the four homologs of chromosomes 1–8 was selected for oligonucleotide design to ensure chromosome-specific oligo probes could cover all four homologous copies of each chromosome. Chromosomes 1–8 of alfalfa were divided into 45 bp oligo sequences using Chorus2 software (https://github.com/zhangtaolab/Chorus2) with default parameters ‘-l 45 -homology 75 -step 5’. Subsequently, oligo sequences from each chromosome were aligned to the genome sequencing data of the alfalfa to filter out repeat-associated oligos. Chromosome-specific oligo sequences were then synthesized *de novo* by MYcroarray companies (Ann Arbor, MI, USA) after adding distinct primer pairs to both ends (Table S2). Finally, chromosome-specific oligo sequences were amplified using primers labeled with FAM (green) or TAMRA (red) to prepare chromosome-specific painting probes according to the published protocol [28]. The rDNA probes used in this study were derived from our previously published article [28].

### Oligo-FISH

The oligo-FISH procedure was performed as previously described [26] with some modifications. The chromosome slides were denatured in formamide solution at 85°C for 5 min, followed by immediate dehydration through an ethanol series (70%, 90%, and 100%) for 5 min each. The hybridization mixture containing 50% formamide, 20% dextran sulfate, 2× SSC (saline-sodium citrate buffer), and 3 μl of oligo probe was applied to the denatured and dried chromosome slides, followed by incubation at 37°C for 12 h. The slides were then washed sequentially with: 2× SSC (room temperature), 2× SSC (42°C, twice), 1× PBS (phosphate-buffered saline, room temperature). Then, the slides were counterstained with 4′,6-diamidino-2-phenylindole (DAPI) (Vector Laboratories, Burlingame, CA, USA; https://vectorlabs.com). Sequential round FISH was performed according to the method described previously [28]. Images were captured using an Olympus DP80 CCD camera (Olympus Corporation, Tokyo, Japan) coupled with an Olympus BX53 fluorescence microscope. FISH images were processed using Image-Pro Plus 6.0 (Media Cybernetics, Inc., Silver Spring, MD, USA) and Adobe Photoshop CC (Adobe Inc., San Jose, CA, USA). For chromosome karyotype analysis, 10 complete mitotic metaphase spreads with no obvious morphological aberrations were selected for arm ratio (AR) = long arm length (L)/short arm length (S), chromosome length (tl) = L + S, relative length (RL) = (tl/total haploid chromosome length [TL] × 100) measurements.

### Divergent time estimation

Phylogenetic analysis was performed using plastome sequences from 16 accessions retrieved from GenBank (Table S4), along with *Trifolium repens* (NC_024036) and *Melilotus albus* (NC_041419) as the outgroup. Sixty-eight coding sequences in all plastomes were concatenated to prepare the alignment, and a maximum likelihood (ML) tree was constructed using iqtree 2.0 [52]. We used MCMCTree software implemented in the PAML4.9e package [53] to estimate divergent times with a codon-partitioned dataset from the concatenated 68 protein-coding genes (PCGs). Due to the lack of reliable fossil calibration points in *Medicago*, we applied two secondary calibration points from Jiao *et al*.: *M. sativa* versus *M. albu*s 12.1–18.5 Mya; *M. albus* versus *T. repens*, 12.2–32.2 Mya [54].

## Supplementary Material

Web_Material_uhaf313

## Data Availability

Data supporting this study are available in the Supporting Information files. The oligo sequences for alfalfa eight chromosome-specific probes are included in Data S1.
