# Supplementary material for: Chromosome-specific painting provides insights into the karyotype evolutionary direction and trajectory in the genus Medicago
Source: Hortic Res. 2025 Nov 14;13(2):uhaf313. doi: 10.1093/hr/uhaf313 (PMC12946675; doi:10.1093/hr/uhaf313)
Supplement: Web_Material_uhaf313 [file web_material_uhaf313.zip › Supplemental Table.docx]

**Table S1.** Details of the eight chromosome oligo pools in alfalfa

| **Alfalfa chromosome** | **FISH probe** | **chromosome length (bp)** | **Total Number of oligos** | **Number of oligos selected** | **Density (oligos/Kb)** | **Primers flanking oligos** |
| --- | --- | --- | --- | --- | --- | --- |
| chr1.4 | chr1 | 88815615 | 191310 | 94675 | 1.07 | P7-P8 |
| chr2.4 | chr2 | 76750018 | 161333 | 95022 | 1.24 | P1-P2 |
| chr3.4 | chr3 | 100414524 | 183095 | 93434 | 0.93 | P5-P6 |
| chr4.2 | chr4 | 93947428 | 181206 | 91418 | 0.97 | P9-P10 |
| chr5.2 | chr5 | 84165483 | 174229 | 91696 | 1.09 | P13-P14 |
| chr6.2 | chr6 | 89579199 | 118104 | 84312 | 0.94 | MF2-MR2 |
| chr7.4 | chr7 | 94657719 | 162327 | 93186 | 0.98 | MF3-MR3 |
| chr8.1 | chr8 | 87242343 | 172010 | 92604 | 1.06 | MF4-MR4 |

**Table S2.** Primer information used in this study.

| **FISH probe** | **Oligo flanking primers F** | **Sequences 5`-3`** | **Oligo flanking primers R** | **Sequences 5`-3`** |
| --- | --- | --- | --- | --- |
| chr1 | P7 | ACTCTCACCTTTACTCCCAC | P8 | CTACTCCCACTACTACCACA |
| chr2 | P1 | TCACCATCCACTCTAAACAC | P2 | CACTTTACACCTCCACTCAT |
| chr3 | P5 | ACTCCCACTCACCTATATCC | P6 | ATAACCTCACTCACCTACCA |
| chr4 | P9 | ATCACACTCCAACTACAACC | P10 | CCCTCACCTCTACACTAAAC |
| chr5 | P13 | TTTACTCGCCCTATATCCAC | P14 | TCAAGTCCCATATCAGCAAG |
| chr6 | MF2 | ATGGGTTTTTACCAGAGCAG | MR2 | TTTGAACGAACACATTACGG |
| chr7 | MF3 | GGCATTCAAGGGAATAACAA | MR3 | TGCCTTTATATGTGCGTGTC |
| chr8 | MF4 | TGGCTCCTTATCTGATCCTC | MR4 | TGATGTGTTTTTGTCGCTTT |

The P-series primers refer to previously published articles by Bi et al. (2019, The Plant Journal)

| **Table S3.** Relative lengths and arm ratios of individual chromosomes in the genus *Medicago.* | | | | | | | | | | | | | | | | |
| --- | --- | --- | --- | --- | --- | --- | --- | --- | --- | --- | --- | --- | --- | --- | --- | --- |
|  | *M. sativa* XinJiangDaYe | | *M. sativa* ZhongMu NO.1 | | *M. varia* GanNong NO.1 | | *M. glutinosa* | | *M. falcata* | | *M. lupulina* | | PI 502447 | | *M. polymorpha* | |
|  | (2n=4x=32) | | (2n=4x=32) | | (2n=4x=32) | | (2n=4x=32) | | (2n=4x=32) | | (2n=4x=32) | | (2n=2x=16) | | (2n=2x=14) | |
| Chr. | Arm ratio^a^ | Relative  Length^b^ (%) | Arm ratio^a^ | Relative  Length^b^ (%) | Arm ratio^a^ | Relative  Length^b^ (%) | Arm ratio^a^ | Relative  Length^b^ (%) | Arm ratio^a^ | Relative  Length^b^ (%) | Arm ratio^a^ | Relative  Length^b^ (%) | Arm ratio^a^ | Relative  Length^b^ (%) | Arm ratio^a^ | Relative  Length^b^ (%) |
| 1 | 1.238±0.025 | 12.449±0.259 | 1.188±0.024 | 12.401±0.24 | 1.213±0.022 | 12.096±0.206 | 1.200±0.025 | 10.900±0.221 | 1.198±0.023 | 11.535±0.371 | 1.188±0.024 | 12.028±0.237 | 1.237±0.033 | 12.639±0.479 | 1.208±0.030 | 13.067±0.369 |
| 2^c^ | 1.196±0.018 | 11.356±0.249 | 1.167±0.016 | 11.582±0.248 | 1.157±0.017 | 10.816±0.163 | 1.138±0.017 | 10.877±0.239 | 1.160±0.019 | 11.224±0.469 | 1.200±0.017 | 11.305±0.261 | 1.159±0.024 | 11.695±0.643 | 1.127±0.025^C^ | 13.391±0.287^C^ |
| 3 | 1.310±0.026 | 12.561±0.241 | 1.330±0.027 | 12.955±0.242 | 1.181±0.017 | 12.803±0.219 | 1.323±0.031 | 12.799±0.289 | 1.416±0.039 | 12.627±0.388 | 1.414±0.029 | 12.979±0.333 | 1.475±0.052 | 14.950±0.484 | 1.122±0.014^A^ | 15.592±0.410^A^ |
| 4^c^ | 1.272±0.028 | 12.720±0.266 | 1.267±0.020 | 12.661±0.234 | 1.256±0.028 | 12.690±0.256 | 1.250±0.040 | 11.503±0.272 | 1.353±0.030 | 14.486±0.498 | 1.441±0.037 | 13.100±0.347 | 1.480±0.042 | 13.784±0.686 | 1.562±0.075^D^ | 17.134±0.783^D^ |
| 5 | 1.213±0.025 | 11.807±0.245 | 1.192±0.023 | 11.815±0.293 | 1.179±0.018 | 10.907±0.203 | 1.126±0.017 | 12.554±0.396 | 1.197±0.018 | 10.907±0.382 | 1.205±0.023 | 11.328±0.223 | 1.150±0.028 | 11.236±0.559 | 1.156±0.032^B^ | 13.230±0.325^B^ |
| 6^d^ | 1.396±0.036 | 14.431±0.226 | 1.478±0.033 | 13.889±0.386 | 1.708±0.033 | 15.703±0.238 | 1.616±0.047 | 18.259±0.553 | 1.642±0.047 | 13.779±0.436 | 1.556±0.037 | 14.673±0.354 | 1.612±0.048 | 15.387±0.438 |  |  |
| 7 | 1.389±0.036 | 12.801±0.285 | 1.273±0.026 | 12.672±0.292 | 1.250±0.021 | 12.456±0.219 | 1.311±0.027 | 11.934±0.296 | 1.345±0.031 | 13.196±0.284 | 1.315±0.029 | 12.448±0.268 | 1.245±0.048 | 9.874±0.464 | 1.258±0.048 | 13.733±0.428 |
| 8 | 1.152±0.017 | 11.875±0.220 | 1.190±0.020 | 12.025±0.273 | 1.214±0.022 | 12.529±0.271 | 1.198±0.023 | 11.174±0.285 | 1.215±0.031 | 12.245±0.279 | 1.310±0.027 | 12.139±0.212 | 1.299±0.028 | 10.435±0.414 | 1.219±0.021 | 13.853±0.340 |

Measurement was performed on each chromosome in 10 metaphase cells. a: Arm ratio, length of the long arm/length of the short arm. b: Relative length, chromosome length/genome length. c: The 5S rDNA locus are located on Chromosome in *Medicago* (x=8). d: The 45S rDNA locus are located on Chromosome in *Medicago* (x=8). A: The newly formed ChrA in *M. polymorpha.* B: The newly formed ChrB in *M. polymorpha.* C: The 5S rDNA locus are located on Chromosome in *M. polymorpha.* D: The 45S rDNA locus are located on Chromosome in *M. polymorpha.*

**Table S4.** Information of 18 accessions used in phylogenetic analysis

| **Species** | **Plant Introduction Number** | **Accession Number** |
| --- | --- | --- |
| *M. monspeliaca* | PI227051 | MK460506 |
| *M. biflora* | PI464827 | MK460504 |
| *M. lupulina* | PI250937 | MK460497 |
| *M. minima* | PI641629 | MK460499 |
| *M. orbicularis* | PI253786 | MK460500 |
| *M. intertexta* | PI498831 | MK460501 |
| *M. laciniata* | PI141474 | MK460503 |
| *M. polymorpha* | PI250782 | MK460498 |
| *M. pironae* | PI253450 | MK460496 |
| *M. arborea* | PI504540 | MK460507 |
| *M. marina* | PI419391 | MK460495 |
| *M. cretacea* | PI631721 | MK460491 |
| *M. sativa* subsp. *glomerata* | PI632028 | MK460494 |
| *M. sativa* subsp. *sativa* | PI516588 | MK460489 |
| *M. sativa* subsp. *falcata* | PI499550 | MK460490 |
| *M. tetraprostrata* | PI577450 | MK460493 |
| *Trifolium repens* |  | NC_024036 |
| *Melilotus albus* |  | NC_041419 |
