# Supplementary figures and images for: Chromosome-specific painting provides insights into the karyotype evolutionary direction and trajectory in the genus Medicago
Source: Hortic Res. 2025 Nov 14;13(2):uhaf313. doi: 10.1093/hr/uhaf313 (PMC12946675; doi:10.1093/hr/uhaf313)

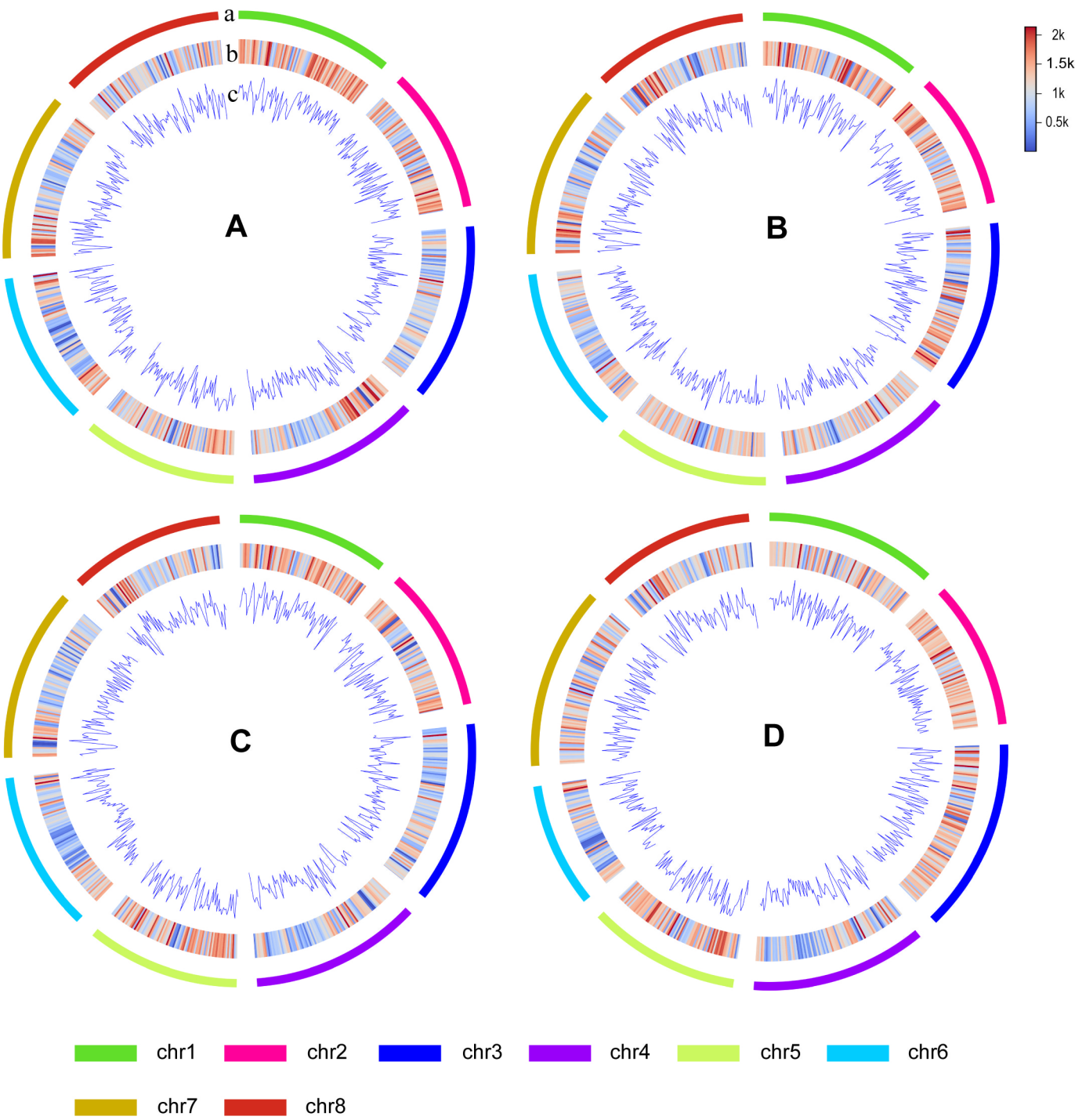

Supplement: Web_Material_uhaf313 [file web_material_uhaf313.zip › Fig S1.pdf]

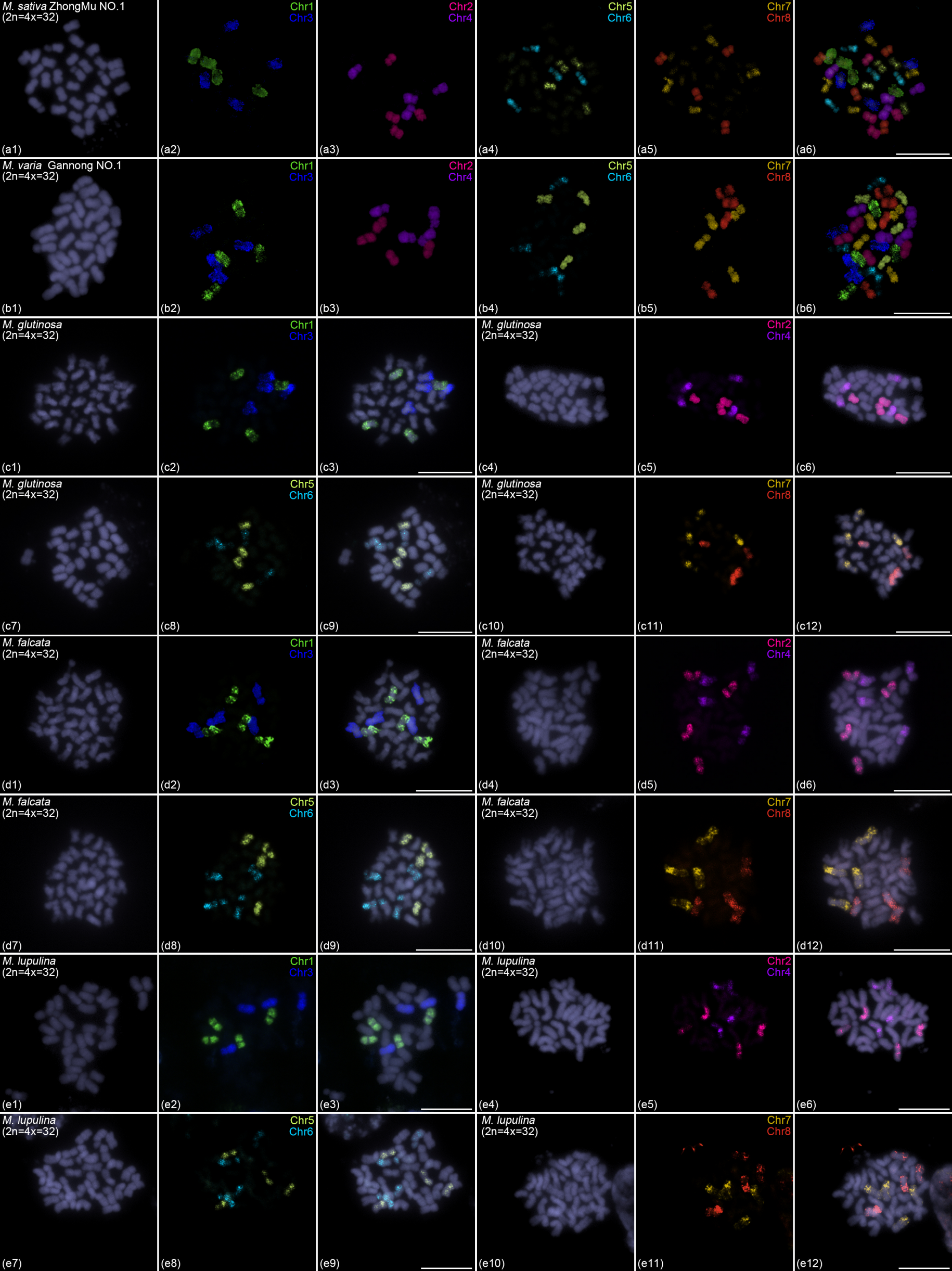

Supplement: Web_Material_uhaf313 [file web_material_uhaf313.zip › Fig S2.pdf]

PI 502447  
(2n=2x=16)

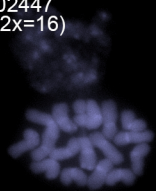

(a1)

Chr1  
Chr3

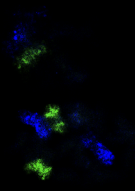

(a2)

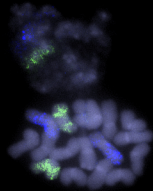

(a3)

PI 502447  
(2n=2x=16)

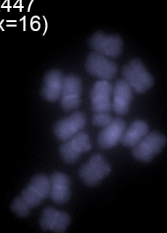

(a4)

Chr2  
Chr4

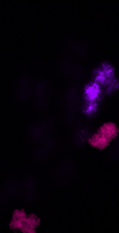

(a5)

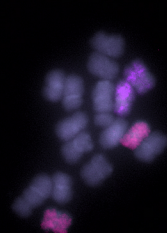

(a6)

PI 502447  
(2n=2x=16)

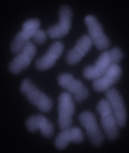

(a7)

Chr5  
Chr6

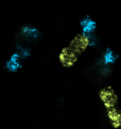

(a8)

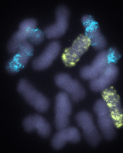

(a9)

PI 502447  
(2n=2x=16)

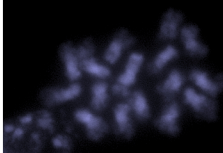

(a10)

Chr7  
Chr8

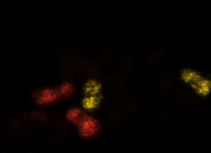

(a11)

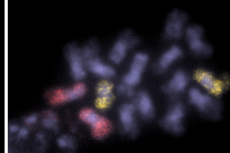

(a12)

Supplement: Web_Material_uhaf313 [file web_material_uhaf313.zip › Fig S3.pdf]

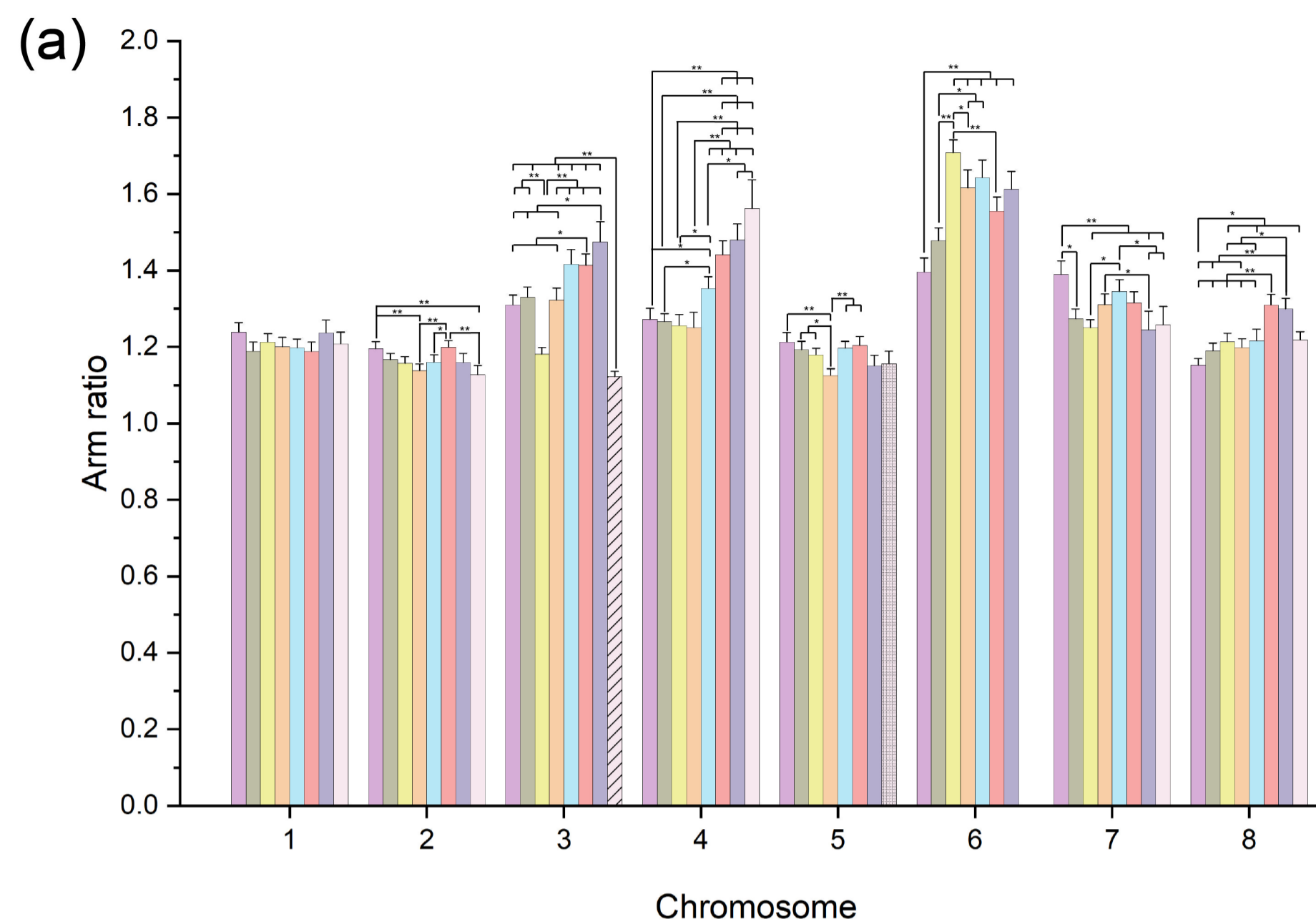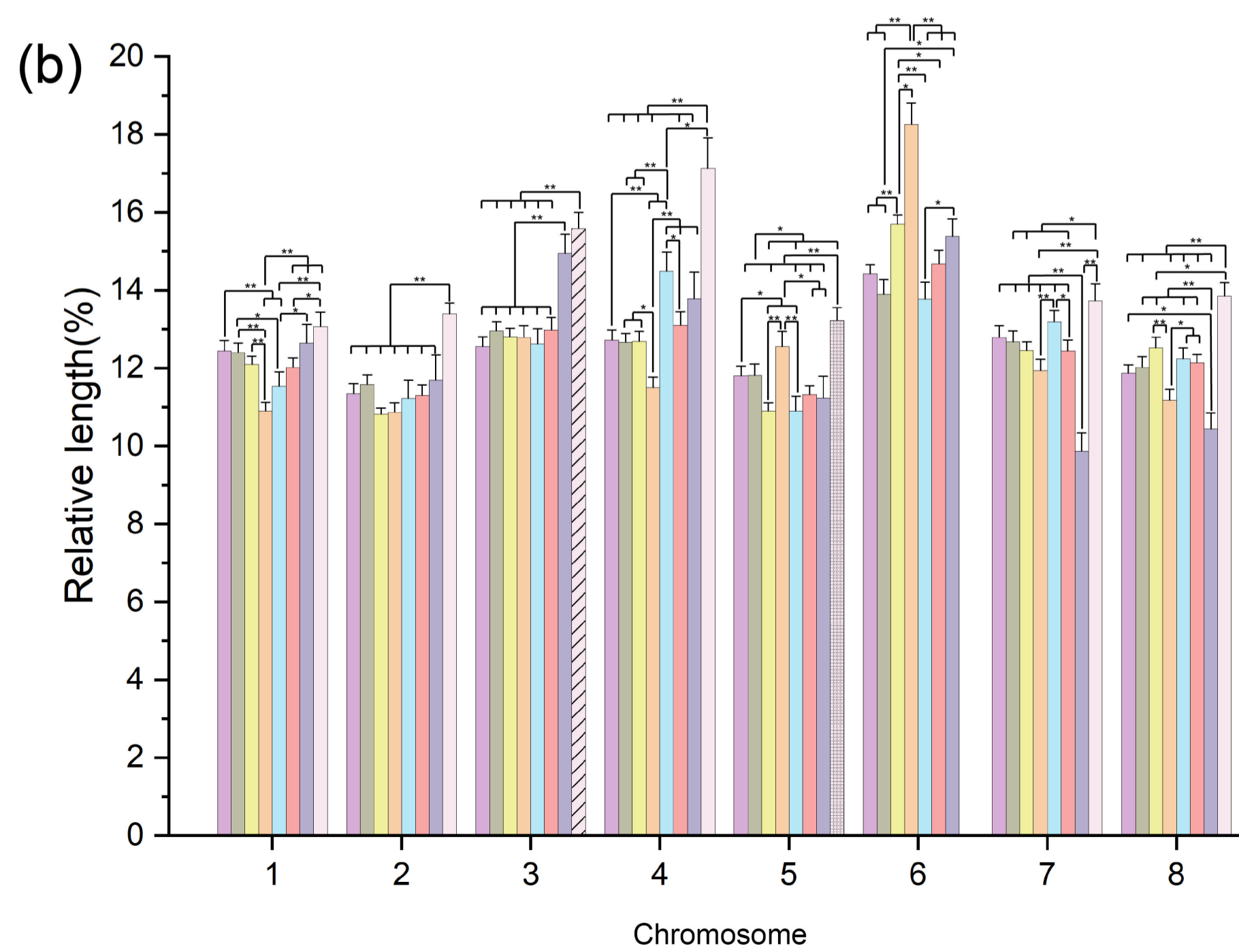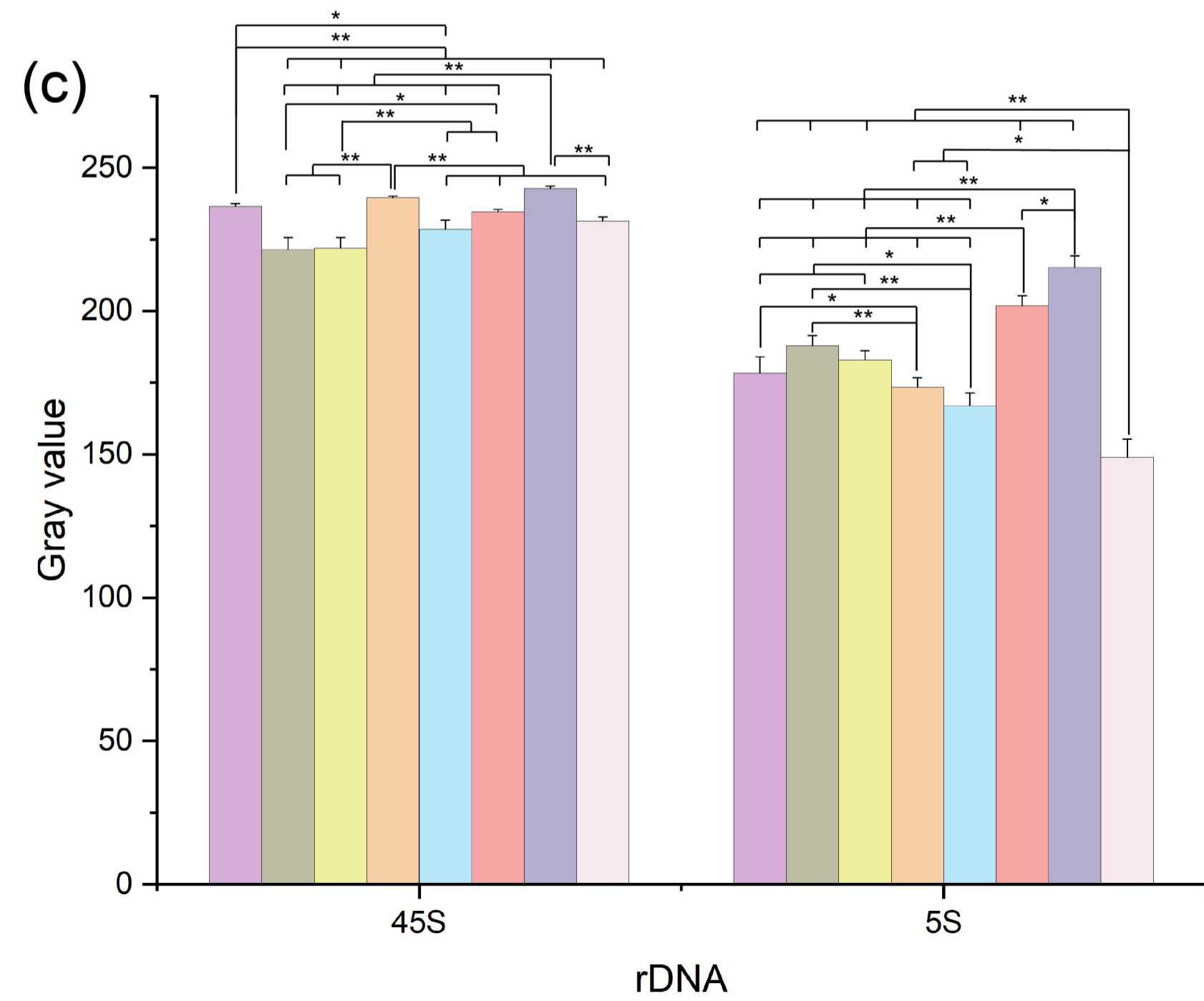

Supplement: Web_Material_uhaf313 [file web_material_uhaf313.zip › Fig S4.pdf]

Chromosome chr6.1

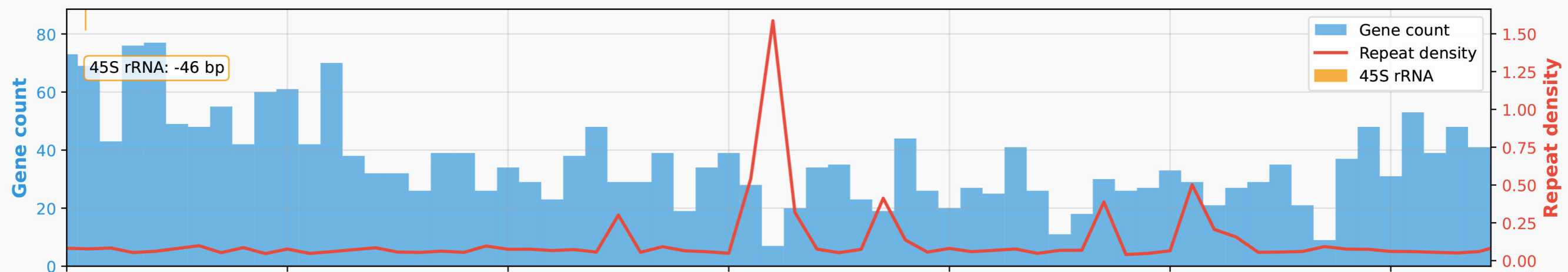

Chromosome chr6.2

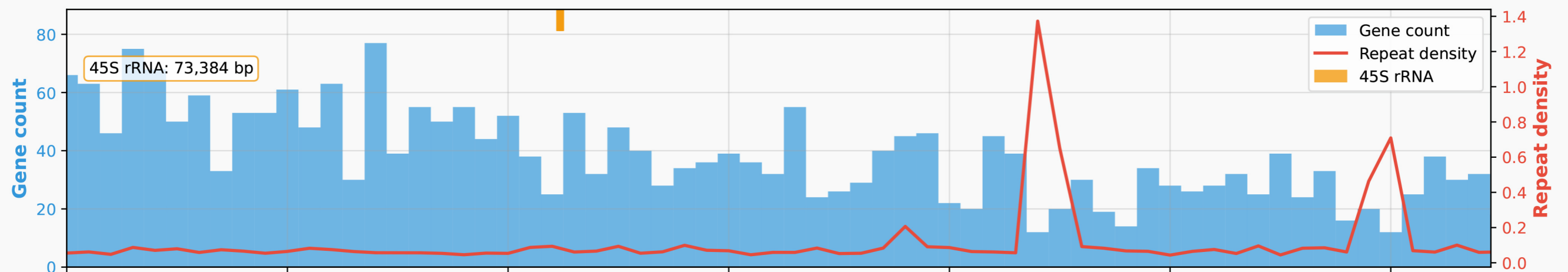

Chromosome chr6.3

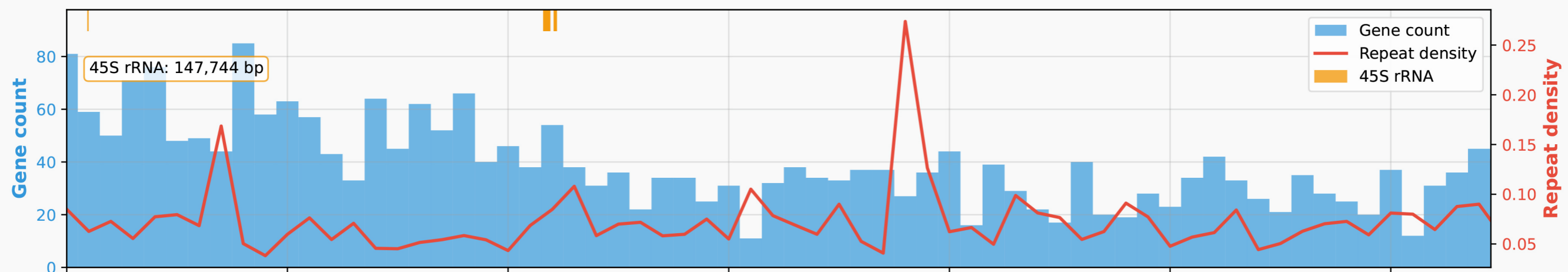

Chromosome chr6.4

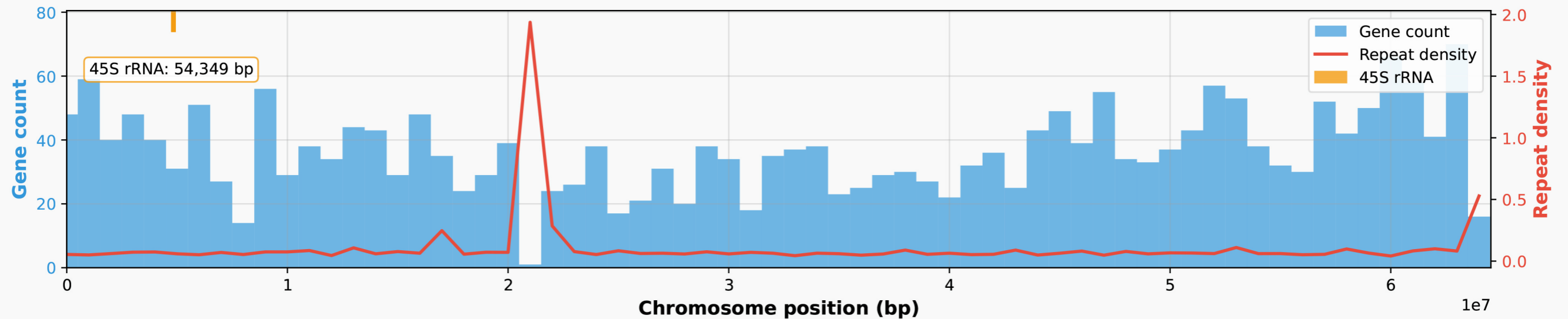

Supplement: Web_Material_uhaf313 [file web_material_uhaf313.zip › Fig S5.pdf]

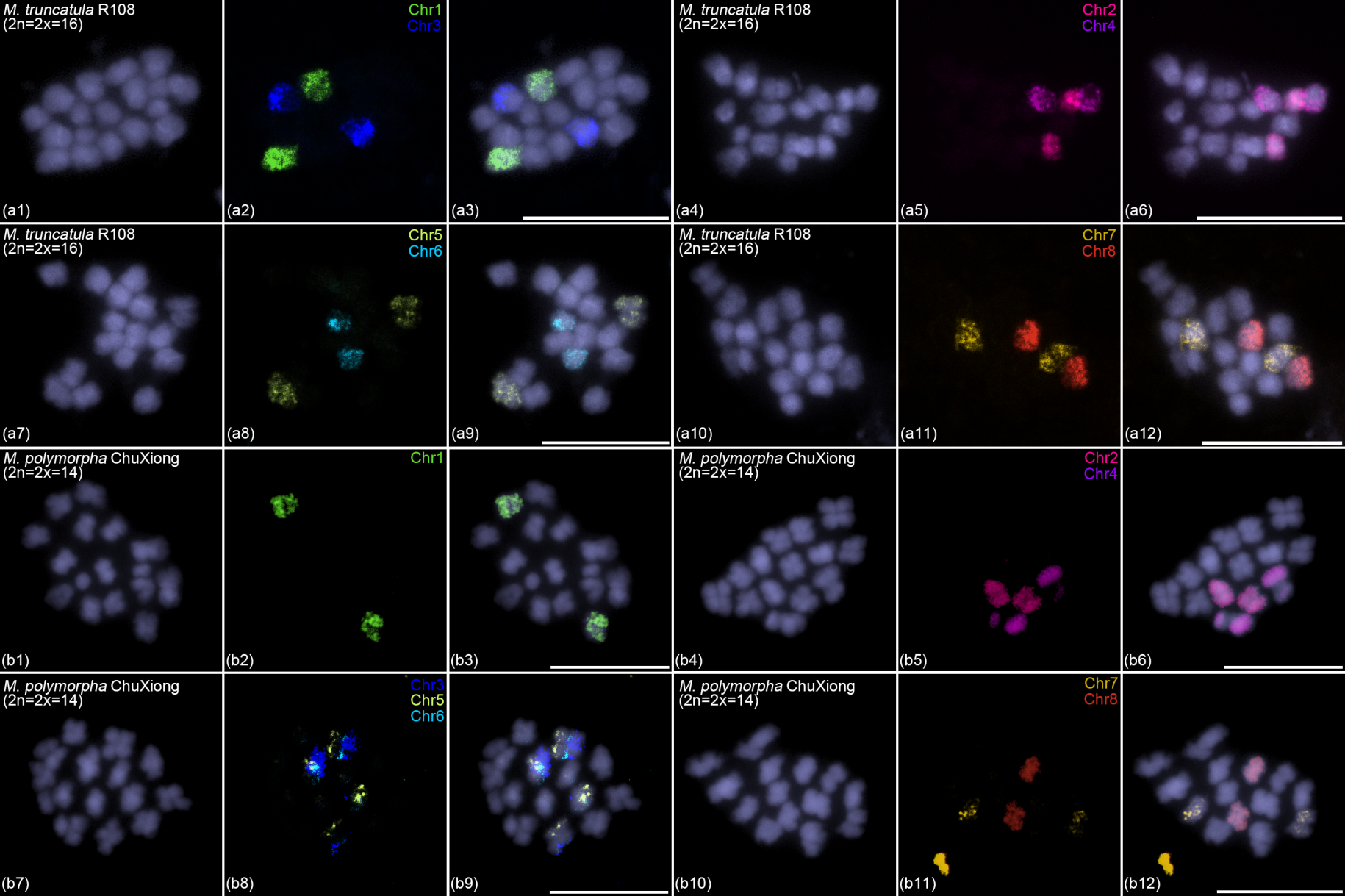

Supplement: Web_Material_uhaf313 [file web_material_uhaf313.zip › Fig S6.pdf]
